# Transcranial Extracellular Impedance Control (tEIC) Modulates Behavioral Performances

**DOI:** 10.1371/journal.pone.0102834

**Published:** 2014-07-21

**Authors:** Ayumu Matani, Masaaki Nakayama, Mayumi Watanabe, Yoshikazu Furuyama, Atsushi Hotta, Shotaro Hoshino

**Affiliations:** 1 Graduate School of Information Science and Technology, The University of Tokyo, Tokyo, Japan; 2 Graduate School of Frontier Sciences, The University of Tokyo, Kashiwa, Japan; University of British Columbia, Canada

## Abstract

Electric brain stimulations such as transcranial direct current stimulation (tDCS), transcranial random noise stimulation (tRNS), and transcranial alternating current stimulation (tACS) electrophysiologically modulate brain activity and as a result sometimes modulate behavioral performances. These stimulations can be viewed from an engineering standpoint as involving an artificial electric source (DC, noise, or AC) attached to an impedance branch of a distributed parameter circuit. The distributed parameter circuit is an approximation of the brain and includes electric sources (neurons) and impedances (volume conductors). Such a brain model is linear, as is often the case with the electroencephalogram (EEG) forward model. Thus, the above-mentioned current stimulations change the current distribution in the brain depending on the locations of the electric sources in the brain. Now, if the attached artificial electric source were to be replaced with a resistor, or even a negative resistor, the resistor would also change the current distribution in the brain. In light of the superposition theorem, which holds for any linear electric circuit, attaching an electric source is different from attaching a resistor; the resistor affects each active electric source in the brain so as to increase (or decrease in some cases of a negative resistor) the current flowing out from each source. From an electrophysiological standpoint, the attached resistor can only control the extracellular impedance and never causes forced stimulation; we call this technique transcranial extracellular impedance control (tEIC). We conducted a behavioral experiment to evaluate tEIC and found evidence that it had real-time enhancement and depression effects on EEGs and a real-time facilitation effect on reaction times. Thus, tEIC could be another technique to modulate behavioral performance.

## Introduction

Electroencephalogram (EEG) recording is a real-time observation of electrophysiological brain activities. The EEG generators are believed to be temporally correlated activities of spatially localized pyramidal neurons (that also share the same orientation). EEG generators in the neocortex are more specifically the sums of excitatory and inhibitory postsynaptic potentials of apical dendrites of pyramidal neurons [1]–[4]. These postsynaptic potentials generate intracellular and extracellular currents (these two currents must electrically continue, i.e., they must be equal at the cell boundary) and a portion of the extracellular currents cause voltage drops on the scalp that are recorded as EEGs. While the dynamics of EEG generators and their networks are nonlinear, the relationship between the generators and volume conduction is linear [4]. Thus, EEG measurements can be treated as voltage measurements of a distributed parameter circuit. Since the typical impedance between two EEG electrodes with an appropriate scalp pretreatment is several kΩ, the amplifiers used for EEG measurements usually provide a high impedance, say 1 MΩ or higher, and do not generate any additional current paths.

Transcranial direct current stimulation (tDCS), as its name suggests, noninvasively stimulates the brain, typically with a 1 mA or higher direct current. Despite it being a simple electric technique, tDCS is believed to have a location-dependent effect on synaptic plasticity. tDCS has been shown to facilitate or inhibit behavioral performance [5]–[16] and to modulate motor evoked potentials (MEPs) [17], [18], EEGs [12], [19], and fMRI signals [20]. Most tDCS studies have employed two electrodes (anodal and cathodal) corresponding to the current polarity. High-definition tDCS, employing one anodal (cathodal) electrode and four surrounding cathodal (anodal) electrodes, was recently proposed for more spatially localized stimulation [21]–[24]. Transcranial random noise stimulation (tRNS), which employs a random noise current of 1 mA order, has been shown to modulate behavioral performance [25] and MEPs [25], [26]. Transcranial alternating current stimulation (tACS), which usually employs a single-frequency sinusoidal current, can modulate and even synchronize with an ongoing brain activity. tACS has been shown to modulate behavioral performance [27]–[30], generate phosphenes [31], and modulate MEPs [27], [32] and EEGs [29], [30], [33]–[36]. tACS variants including phase variations [30], [34] and DC offsets [27], [29] have been proposed. tACS has complicated location-, frequency-, and phase-dependent effects [37]. Although the behavioral and physiological effects of these stimulations are usually evaluated in a before-and-after manner, they are sometimes treated in real-time [14]–[16], [28], [30], [34]. A real-time current stimulation, or local field potential guided current stimulation as feedback, was recently proposed [38]. Needless to say, all these stimulations involve attaching artificial electric sources to the scalp or brain area.

We came up with a simple idea; what would happen if the electric source that would normally be attached to the scalp were replaced with a low-impedance resistor? It would initially decrease the extracellular resistance and then increase the volume current or extracellular current; i.e., it would also increase the intracellular current. In turn, it would increase postsynaptic potentials and might modulate behavioral performance. In this paper, we describe a study to control extracellular impedance by attaching a negative resistor to the scalp. We describe the electric theory involved and EEG simulations of this technique. After that, we show actual EEGs and behavioral performances when using it.

## Materials and Methods

### Basic Concept of tEIC

We present a new electric technique to noninvasively modulate extracellular currents and behavioral performances, called transcranial extracellular impedance control (tEIC). The basic concept of tEIC is as follows.

**Figure 1:**
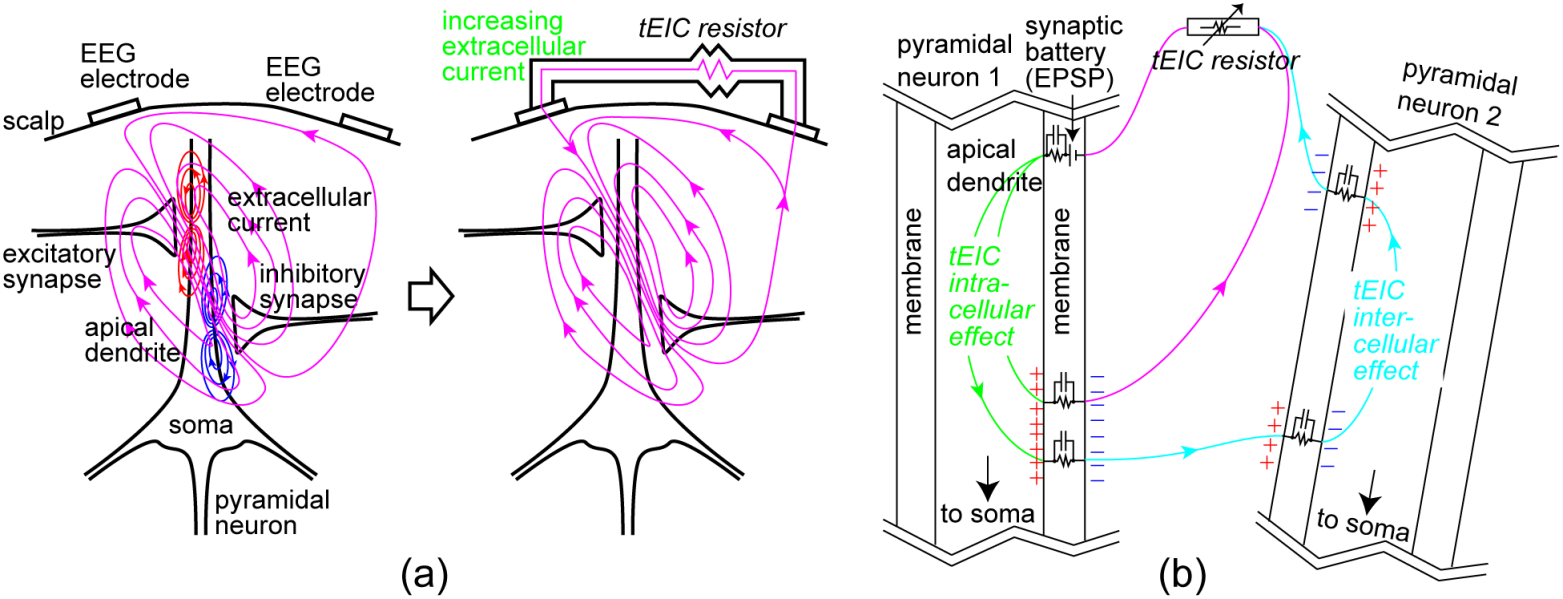
Concept of tEIC. (a) Left: EEGs are voltage drops of extracellular currents, the origins of which are dendric currents of pyramidal neurons, between the EEG electrodes; Right: When a transcranial extracellular impedance control (tEIC) is applied, i.e., a shunt resistor is attached to two EEG electrodes to reduce the extracellular resistance, the extracellular currents increase, and the intracellular currents increase accordingly because these two currents are continuous; i.e., they must be equal at the cell boundary. (b) Left: Effects on pyramidal neuron 1. It is called the tEIC intracellular effect when the intracellular current modulation of a pyramidal neuron is caused by the current from the neuron itself. The increasing currents increase the voltage drop, and the membrane potential increases. Right: Effects on pyramidal neuron 2. It is called the tEIC intercellular effect when the modulation is caused by currents from other neurons. tEIC intra- and inter-cellular effects nearer the soma have a greater effect on firing. Note that the + and – marks show only the effects of intra- and inter-cellular currents on membrane potentials neglecting the resting potential, and the current branches that do not pass through the tEIC resistor are omitted.

As described in the introduction, EEGs are generated by taking the sum of spatiotemporally localized excitatory and inhibitory postsynaptic potentials (EPSPs/IPSPs) (left panel in Fig. 1(a)). If tEIC is applied, i.e., a shunt resistor is connected to the two EEG electrodes, and the extracellular resistance decreases and extracellular current increases accordingly (right panel in Fig. 1(a)). The intracellular current also increases because of the current continuity. We call this the tEIC intracellular effect. The tEIC intracellular effect increases the voltage drop, and therefore, the membrane potentials also increase as shown in pyramidal neuron 1 of Fig. 1(b). This is our hypothesis based only on Ohm’s law. However, there was a simulation study in which the extracellular impedance had an influence on membrane potentials [39].

On the other hand, a pyramidal neuron has currents passing through it from other pyramidal neurons (see pyramidal neurons 1 and 2 in Fig. 1(b)). These passing currents not only increase, but also decrease with tEIC depending on the relative locations of the pyramidal neurons. For instance, if tEIC pulls out a portion of the current originating from pyramidal neuron 1 flowing into 2, the passing current decreases for pyramidal neuron 2. We call this the tEIC intercellular effect. The tEIC intercellular effect also increases or decreases the membrane potentials, although the inward currents and outward currents have opposite effects on them. The effect is not necessarily random and may consistently enhance or depress the membrane potentials because the pyramidal neurons in the neocortex are spatially localized not only in their location but also in their orientation. This is also our hypothesis based only on Ohm’s law. However, there were in vitro studies [40], [41] and a simulation study [42] in which a portion of the current from an active neuron influenced the membrane potentials of other neurons via extracellular impedance. In any case, the tEIC intra- and inter-cellular effects nearer the somas have more influence on firing in real-time. Thus, they might also affect behavioral performance in real-time.

A shunt resistor connection with contact resistances lying between the two EEG electrodes and the scalp may exhibit only limited tEIC intra- and inter-cellular effects. If the resistor is, however, replaced with a negative resistor, which can cancel the contact resistances and goes into the negative resistance domain, theoretical and experimental investigations of tEIC would be worth considering.

### Electric Circuit Theorems’ Perspective

**Figure 2:**
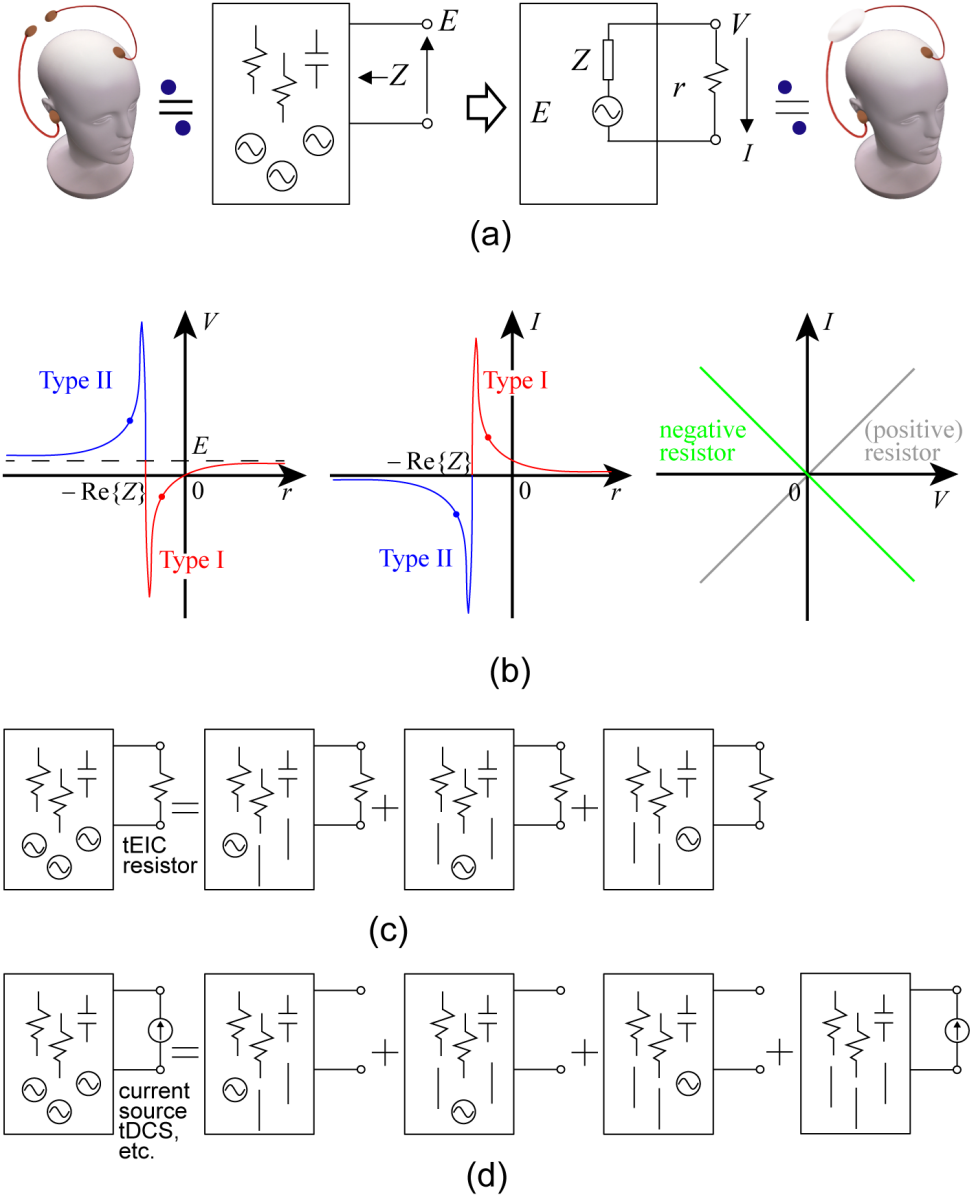
Operating principle of tEIC. (a) According to Ho-Thevenin’s theorem, a head including the brain as seen from two EEG electrodes can be electrically approximated by a very simple circuit. (b) Left and middle: The resistance versus voltage and resistance versus current characteristics are calculated on the basis of the theorem. These characteristics are symmetric with respect to the points [–Re{*Z*}, *E*] and [–Re{*Z*}, 0]. Thus, Type I tEIC positively increases (or enhances) the current *I* (a portion of the extracellular currents), and Type II tEIC “negatively” increases (or depresses) it. Right: The negative resistor here is an Ohmic resistor having a negative slope. (c) The superposition theorem resolves the head circuit under tEIC into a number of circuits, each including one active EEG generator (the others are replaced with a short circuit) and the tEIC (a Type I or Type II resistor). Each resolved circuit indicates that a tEIC resistor enhances or depresses each active EEG generator. This is the tEIC intracellular effect. (d) It also demonstrates that a current source (e.g., tDCS, tRNS, and tACS) can stimulate a bunch of EEG generators (actually replaced with a short circuit) regardless of whether each of them is active.

Although the dynamics of neurons and neural networks are nonlinear, an EEG observation, which consists of a volume conductor involving EEG generators, is linear. The EEG forward model is, indeed, expressed as a superposition of EEG generator activities. The left panel in Fig. 2(a) shows a linear black-box circuit approximating the head and brain for an EEG observation and its equivalent circuit given by Ho-Thevenin's theorem [43]. The theorem is described as follows. From any black-box circuit having passive devices (resistors, inductors, and capacitors) and electric sources, one can see a voltage *E* (time-varying) and an impedance *Z* between the electrodes. For the calculation of *Z*, all the electric sources are killed. “Killed” is a legitimate technical term meaning that each voltage source is replaced with a short circuit, whereas each current source is replaced with an open circuit. So long as the circuit remains a black-box from the point of view of the electrodes, it will be equivalent to a simple circuit having a voltage source *E* and internal impedance *Z*. Thus, when a shunt resistor *r* is attached to the EEG electrodes (right panel in Fig. 2(a)), the resistance *r* versus voltage *V* and *r* versus current *I* characteristics can be calculated as *V* = *rE*/(*Z*+*r*) and *I* = *E*/(*Z*+*r*). The left and middle panels in Fig. 2(b) show the real parts of these characteristics. We would like to stress here that *I* and *V* vary in accordance with the real-time EEG *E*. Note that the negative resistors are ones having a negative slope in their *V*-*I* characteristics as shown in the right panel in Fig. 2(b), and although negative resistors that simply obey Ohm's law do not exist per se, we can use operational amplifier circuits (explained later) instead.

The *r*-*V* and *r*-*I* characteristics are symmetric with respect to the points [–Re{*Z*}, *E*] and [–Re{*Z*}, 0], respectively (Fig. 2(b); Re: the real part of *Z* in parentheses). We call *r* = –Re{*Z*} the separator. If Im{*Z*} = 0 (Im: the imaginary part of *Z* in parentheses), the separator will be a pole. We have two types of extracellular impedance control across the separator; Type I: to increase the real-time postsynaptic current, and Type II: to decrease the real-time postsynaptic current. The control gain can be set by selecting the resistance *r*. The actual operating points (blue and red dots) are chosen to be located on hyperbolical-like curves. Note that we are only concerned, throughout this paper, with the left and right hyperbolical-like curves, not the middle steep connection between the curves.

How does tEIC locally affect each EEG generator in the brain? Since the brain model is linear, the superposition theorem [43] also holds, as follows (Fig. 2(c)). The circuit on the left-hand side of the figure is expressed by the sum of the right-hand side circuits, in each of which one electric source is kept alive and the others are killed. A positive shunt resistor connection makes the resistance (or extracellular resistance) seen from each voltage source (or each EEG generator) decrease because the connection is parallel, and it provides an additional current path. Each voltage source thus increases the current in whose direction it applies. Now, if the positive shunt resistor is replaced with a negative shunt resistor in the Type I domain, the current more positively increases than it would with a positive resistor, as shown in the *r*-*I* characteristic in the middle panel of Fig. 2(b). Moreover, if it is replaced with a negative shunt resistor in the Type II domain, the absolute value of the current in the negative domain increases. These modulation (Type I: increase and Type II: decrease) effects of the current (or intracellular current) constitute the tEIC intracellular effect.

The superposition theorem accounts for the difference between tEIC and other stimulations such as tDCS, tRNS, and tACS. In Fig. 2(d), a current source instead of a resistor is attached to two EEG electrodes (usually to two electrode sponge pads) used to apply the current stimulations. The circuit on the right-hand side of the figure is resolved into the four circuits on the left-hand side. The sum of three out of four of these circuits on the left is equivalent to just an EEG recording without stimulation. The stimulation effect is expressed by only the right-most circuit having all the killed EEG generators. The current stimulations work independently of the activity of each EEG generator and moreover independently of whether or not each EEG generator is active. This is the essential difference between using a resistor or an electric source. If, however, one would like to stimulate EEG generators independently of their activities, there is nothing that can be done because tEIC is just a resistor.

### Electronics for tEIC

**Figure 3:**
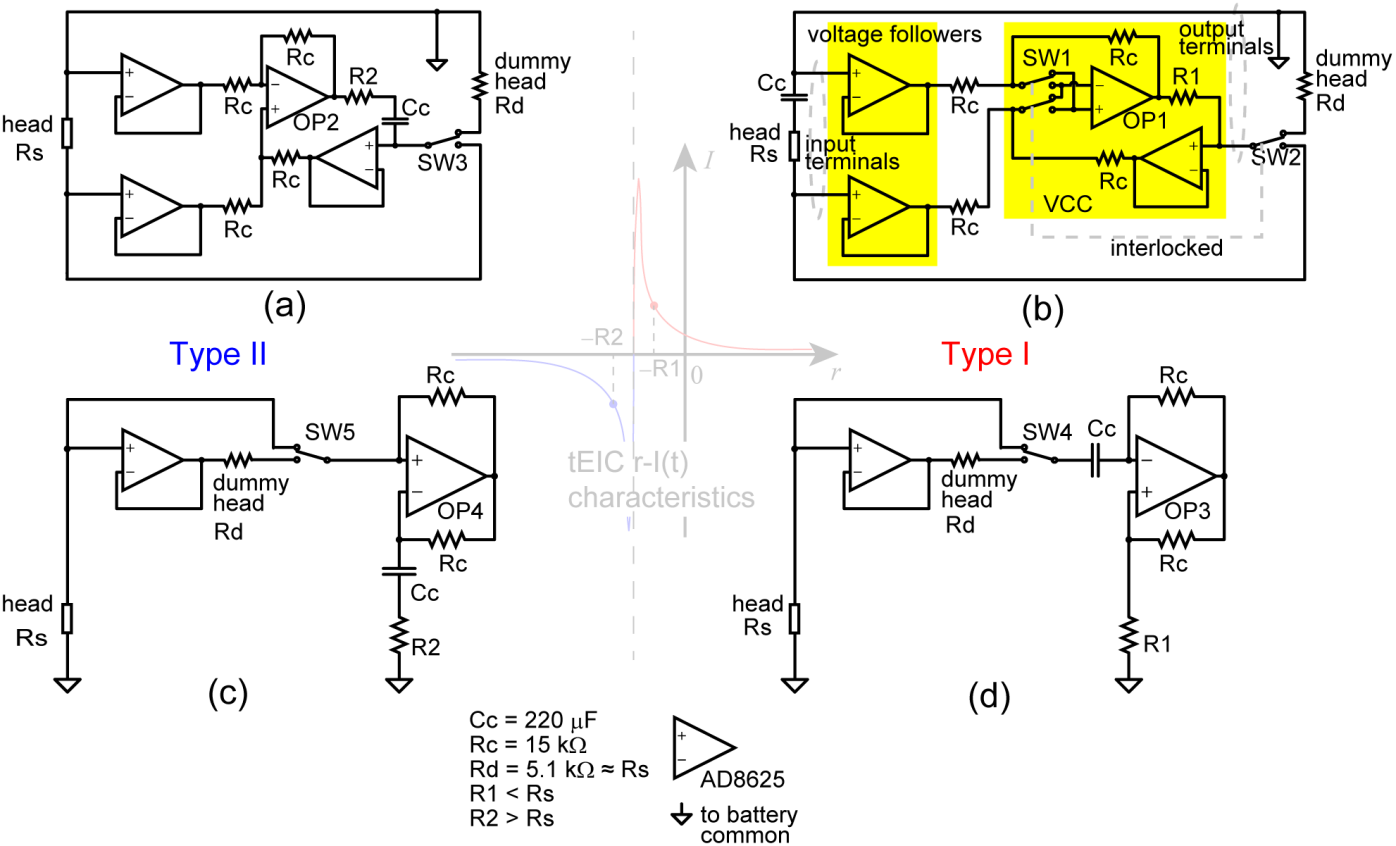
Electronics for tEIC. The implementations of Type I tEIC (positive amplification of extracellular currents) and Type II tEIC (negative amplification of them) are different from each other, and two implementations for each type are illustrated. (a) and (b) are respectively implementations of tEIC with two voltage followers and a voltage-to-current convertor (VCC) for Type II and Type I tEICs, respectively. (c) and (d) are implementations with a voltage follower and a common negative impedance circuit. Every circuit has a switch (SW1 and SW2 are interlocked) for choosing two conditions; one is for the tEIC condition, i.e., applying the current to the brain and the other is for the sham condition, i.e., applying the current to a dummy head resistor. The device parameters are listed at the bottom of this figure; the Type I (Type II) implementation functioned as –R1 Ω (–R2 Ω) above 4 Hz without a phase delay.

Most of the Type I tEICs and all of the Type II tEICs work in the negative resistance domain (left and middle panel in Fig. 2(b)). The type I tEIC in the positive resistance domain is relatively less effective because it is distant from the separator, and hence, its connection would only result in a small modulation. Thus, Type I and Type II tEICs should be realized with negative resistors as a first tEIC application. Figures 3(b) and 3(a) show negative resistors, i.e., –R1Ω for Type I tEIC and –R2Ω for Type II tEIC (assuming that –R2 < separator (–Rs) < –R1); both resistors are actually operational amplifier circuits. Each circuit consists of two voltage followers and a voltage-to-current convertor (VCC) [44]. For the tEIC conditions, the input terminals of the voltage followers and the output terminals of the VCC are each connected to the head via EEG electrodes. Thus, the VCC negatively feeds back the current to the brain according to the real-time EEG voltage and the voltage followers avoid reentering the current into the VCC, so the overall configuration works as a negative resistor. Switches SW1, SW2, and SW3 are used to switch experimental conditions (e.g., Type I, Type II, and Sham). For the Sham condition, which is a placebo, the output terminals of the VCC are connected to a dummy head resistor in both circuits.

Although it is not of scientific concern, the Type I and Type II tEIC circuits (Figs. 3(b) and 3(a)) are slightly different from each other so that the operational amplifiers OP1 and OP2 can stably operate. OP1 and OP2 have positive and negative feedback connections. For stable operation, the inverted and noninverted input terminals of OP1 and OP2 must be set such that the negative feedback is greater than the positive one. The magnitude of the feedback is determined by the resistance of the head, Rs, and R1 or R2. Therefore, in Type I circuits (Fig. 3(b)), the connections of these two input terminals must be swapped depending on whether the output terminals of the VCC are connected to the head or the dummy head (thus, SW1 and SW2 are interlocked). Such tricky swaps are not necessary in Type II circuits (Fig. 3(a)). In addition, the locations of the coupling capacitors Cc, which work as high-pass filters, are also different from each other to ensure stable operation (note that the DC impedance of the capacitors is infinity). The actual values of the coupling capacitors are experimentally determined such that they pass the theta band and higher (say, >4 Hz) without a phase delay. This is because we are interested in the so-called AC EEG rather than the DC EEG (DC and 1/f fluctuation components). The switching between the head and dummy head is to keep these capacitors charged to reduce switching transients in the real-time EEG. The common forms of negative resistors [45] shown in Figs. 3(c) and 3(d) can be used instead of the circuits shown Figs. 3(a) and 3(b). Workarounds also exist to ensure stable operation of the electronics in Fig. 3 (see Text S1).

A detailed description of the tEIC electronics would be complicated and of little use to those who are interested in using tEIC in their behavioral research. The electronics in Fig. 3 are to just what is needed to implement a negative resistor whose voltage and current characteristic as shown in the right panel in Fig. 2(b). The electronics for tDCS, tRNS, and tACS would be equally complicated since they must also have a VCC. Electronics engineers/technicians can easily build tEIC electronics by soldering together the various components, but they must take care of the difference between Type I and Type II. When one would like to attach tEIC electronics to EEG electrodes, s/he must first measure the resistance between the electrodes with an impedance check function that any commercial EEG amplifier would provide. The resistance is a sign-inverted separator value. S/he should start from the separator +5 kΩ for Type I electronics and –5 kΩ for Type II and then get closer to the separator if necessary by monitoring the EEG in real-time. A big voltage jump will be observed just after switching the electronics (switching transients), but the level will gradually come back to zero. This is due to the initial charge of the coupling capacitors. S/he may occasionally find big voltage jumps around, say, ±2 kΩ or closer to the separator whenever switching. This is due to the limits of the electronics, and therefore, an additional resistance margin from the separator must be added.

Let us turn to the matter of safety. If an accident happens to the electronics during tEIC, the operational amplifiers would output the voltage for driving. The tEIC circuits are always battery-driven (±3 V with four AA batteries), and thus, the subjects would be stimulated with a 1-mA-order current (comparable to existing current stimulations) in the worst case, since Rs is typically several kΩ. In practice, we can set the current flowing through the tEIC resistor to be of 10 nA order. This allows us to make EEG recordings (with a non-descript EEG amplifier) and perform tEIC simultaneously under the usual EEG amplifier settings (e.g., gains and filters) without having to learn any tricky EEG recording techniques (e.g., gating).

## Simulations of tEIC

### Methods

The operating principle of tEIC based on the superposition theorem has been shown to yield the tEIC intracellular effect in the Electric Circuit Theorems’ Perspective subsection; i.e., Type I enhances and Type II depresses the intracellular current of each EEG generator. We performed three simulations A–C to see how the tEIC intercellular effect occurs. Two channels to which tEIC was attached (tEIC channels, hereafter) were different from one simulation to another. One of the tEIC channels was an indifferent reference only in Simulation A. Here, we describe only Simulation A (see Text S2 and Fig. S1 for descriptions of Simulations B and C).

**Figure 4:**
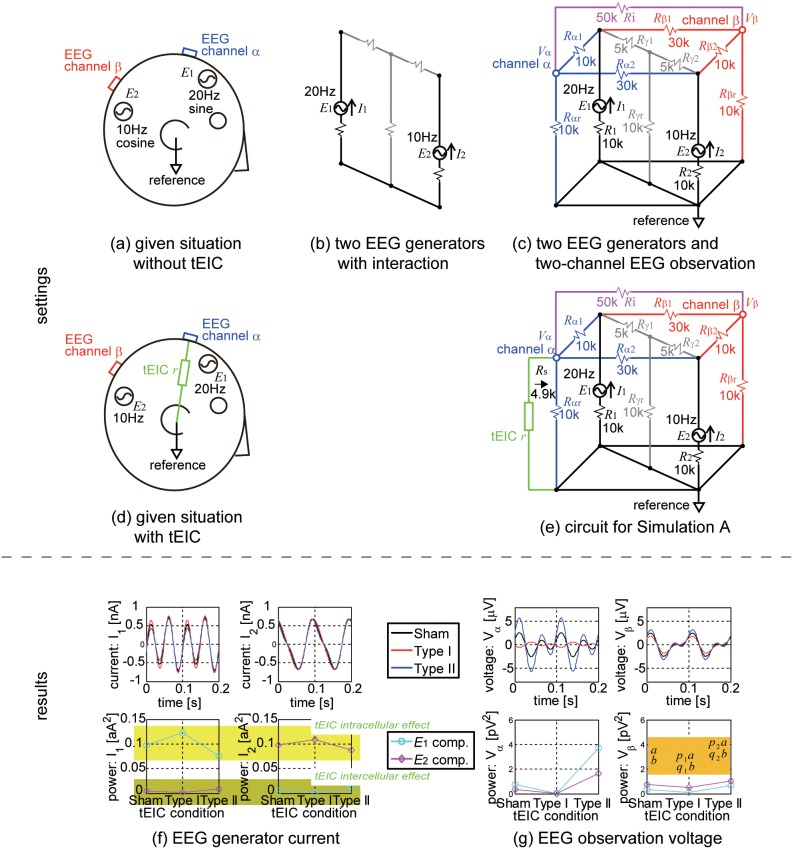
Simulation A. (a) Two EEG generators *E*_1_ (20 Hz sine) and *E*_2_ (10 Hz cosine) were in the brain, and a two-channel (α and β) EEG was observed (indifferent reference: right earlobe). (b) The basic mutual interaction between the EEG generators was given by the gray T-circuit. (c) The EEG observation on channel α (β) was given by the blue (red) T-circuit bifurcated from the gray T-circuit. The direct path between channels α and β was given by the magenta connection. The bottom surface of the cubic circuit was connected to the reference. Resistors were set such that channel α (β) was more sensitive to *E*_1_ (*E*_2_). (d) tEIC resistor *r* was connected to EEG channel α and the reference. (e) Circuit for Simulation A. (f) tEIC-conditional current waveforms of the EEG generators (top row) and their powers resolved into *E*_1_- and *E*_2_-originated currents (bottom row). Type I enhanced and Type II depressed the intrinsic currents (*E*_1_-originated current in *I*_1_ and *E*_2_-originated current in *I*_2_) compared with the Sham condition. This is the tEIC intracellular effect. Type I depressed and Type II enhanced the interference currents (*E*_2_-originated current in *I*_1_ and *E*_1_-originated current in *I*_2_) compared with the Sham condition. This is the tEIC intercellular effect. Thus, the synergetic tEIC effect clearly occurred; i.e., Type I differentiated and Type II merged the EEG generator activities. (g) tEIC-conditional voltage waveforms of the EEG observations (top row) and their powers resolved into *E*_1_- and *E*_2_-originated voltages (bottom row). The results in the top left panel show that the tEIC-conditional waveform changed on the basis of Ho-Thevenin’s theorem.

We assumed that two EEG generators *E*_1_ and *E*_2_ were located in the brain and two-channel EEG (channels α and β) were measured with an indifferent reference of the right earlobe (Fig. 4(a)). *E*_1_ and *E*_2_ had basic mutual current interference via the gray T-circuit (Fig. 4(b)). The EEG observation at channel α (β) was enabled by the blue (red) T-circuit bifurcated from the gray T-circuit (Fig. 4(c)). Channels α and β also had a direct path with the magenta connection. Consequently, all these connections contributed to mutual current interference between *E*_1_ and *E*_2_. The bottom surface of the cubic circuit was connected to the reference. *E*_1_ and *E*_2_ were respectively driven with 10sin(2π20*t*) and 10cos(2π10*t*) µV (*t*: time) for separately analyzing *E*_1_- and *E*_2_-originated components and were connected with the reference via resistances *R*_1_ = 10 kΩ and *R*_2_ = 10 kΩ. The above-mentioned settings were common to all the simulations. In Simulation A, the other resistors were set as follows; basic interference: *R*_γ1_ = 5 kΩ, *R*_γ2_ = 5 kΩ, and *R*_γr_ = 10 kΩ; channel α: *R*_α1_ = 10 kΩ, *R*_α2_ = 30 kΩ (high sensitivity to EEG generator *E*_1_); channel β: *R*_β1_ = 30 kΩ, and *R*_β2_ = 10 kΩ (high sensitivity to EEG generator *E*_2_); and the direct path between channels α and β: *R*_i_ = 50 kΩ.

tEIC resistor *r* was set as the separator +4 kΩ for Type I tEIC and the separator –4 kΩ for Type II tEIC. Each separator (–*R*_s_) was the sign inversion of the inner resistance (*R*_s_) seen from each tEIC resistor. *R*_s_ was numerically calculated. This was a common setting in all the simulations. In Simulation A, the tEIC channels were channel α and the reference (Figs. 4(d) and 4(e)). Since *R*_s_ was 4.9 kΩ, the Type I and II resistors were set as –0.9 kΩ and –8.9 kΩ, respectively.

### Results: Simulation A

First, let us describe how tEIC affects the EEG generators. The current waveforms *I*_1_ and *I*_2_ flowing through *E*_1_ and *E*_2_ tEIC-conditionally differed from one another (top panels in Fig. 4(f)) because of the differences between the resolved currents originating from *E*_1_ and *E*_2_ (bottom panels in Fig. 4(f)). Type I enhanced and Type II depressed the intrinsic currents (the *E*_1_-originated current in *I*_1_ and the *E*_2_-originated current in *I*_2_). This is evidence of the tEIC intracellular effect. On the other hand, Type I depressed and Type II enhanced the interference currents (the *E*_2_-originated current in *I*_1_ and the *E*_1_-originated current in *I*_2_). This is evidence of the tEIC intercellular effect. Moreover, we would like to mention three things. First, these tEIC intra- and inter-cellular effects were more prominent for *E*_1_ than for *E*_2_. That is, they are functions of the sensitivities to the EEG generators, or the inverse of the resistances. Second, the two effects were synergistic on the EEG generators. That is, Type I differentiates, whereas Type II merges the EEG generator activities. We call this the synergetic tEIC effect. Third, the tEIC effects were, albeit trivial because of the linear electric circuits, time- and phase-locked to the Sham condition. That is, tEIC only affected the active EEG generators. To be precise, the tEIC intracellular effect only occurred on the active EEG generators.

Next, let us describe how the tEIC affects the EEG observations. The EEG of channel α, *V*_α_, indicated an ascending absolute magnitude order: Type I ≤ Sham ≤ Type II, and a polarity inversion only for Type I (top left panel in Fig. 4(g)). These results are consistent with Ho-Thevenin’s theorem. The Type I polarity inversion was caused by the fact that the parallel combined resistance of *R*_αr_ and *r* became negative only for Type I. On the other hand, the EEG of channel β, *V*_β_, showed the same magnitude order but no conditional polarity inversion (top right panel in Fig. 4(g)). The polarity stayed the same under the different parameter settings, and the order was rarely hard to distinguish. Thus, one can quickly determine whether or not the tEIC works properly by seeing that the Type I polarity inversion only occurs in the tEIC channels. If the Type I polarity inversion occurs in other channels, tEIC could be a current stimulation instead. The current stimulation must propagate from the tEIC channel. Since the tEIC circuits in Figs. 3(a) and 3(b) included a VCC, perhaps it acts as a current stimulator for some reason.

### Discussion: Simulation A

*V*_β_, which was not a tEIC channel, reflected the synergetic tEIC effect as follows. If the synergetic tEIC Type I (Type II) effect occurs, each EEG generator is more differentiated from (merged with) each other in comparison with the Sham condition. Thus, Type I (Type II) seems to enhance (depress) the spatial resolution of the EEG generators. This appears in the EEG as if Type I (Type II) has worked as a spatial high-pass (low-pass) filter. Let *a* and *b* respectively denote the *E*_1_- and *E*_2_-originated components in the power of *V*_β_ for the Sham condition, as shown in the inset of the bottom right panel in Fig. 4(g). Type I (Type II) multiplies *a* by *p*_1_ (*p*_2_) and *b* by *q*_1_ (*q*_2_) to modulate these components. The normalized power of the *E*_1_-originated component in *V*_β_ of the Sham condition is *a*/(*a*+*b*) and that of Type I is *p*_1_ *a*/(*p*_1_ *a*+*q*_1_ *b*). The ratio of these two powers is *p*_1_(*a*+*b*)/(*p*_1_ *a*+*q*_1_ *b*). For the *E*_2_-originated component, this ratio is *q*_1_(*a*+*b*)/(*p*_1_ *a*+*q*_1_ *b*), accordingly. Thus, these two ratios are *p*_1_/*q*_1_ independently of *a* and *b*, namely the amplitudes of *E*_1_ and *E*_2_. In the case of Type II, the ratio is *p*_2_/*q*_2_. In this simulation, these values were calculated as *p*_1_/*q*_1_ = 1.9>1 and *p*_2_/*q*_2_ = 0.7<1. Although the large/small relations between these values and 1 were inverted in the case that the EEG sensitivities changed such that channel β was more sensitive to *E*_1_ than to *E*_2_, *q*_1_/*p*_1_ and *q*_2_/*p*_2_ should be used for the evaluation in place of *p*_1_/*q*_1_ and *p*_2_/*q*_2_. In any case, the large/small relations indicated that Type I (Type II) had higher (lower) sensitivity to nearby EEG generators and lower (higher) sensitivity to distant EEG generators in comparison with the Sham condition. Although the sensitivity depends on the electrical distance, which does not necessarily correspond to the Euclidean distance, it is quite natural that EEGs have high (low) sensitivity to nearby (distant) EEG generators; i.e., the situation of this simulation is illustrated in Fig. 4(d). Therefore, Type I (Type II) could act as a spatial high-pass (low-pass) filter through the synergistic tEIC effect.

## Behavioral Experiment with tEIC

### Materials and Methods

We performed a behavioral experiment to discover the tEIC intra- and inter-cellular effects on EEGs and to find an answer to a simple question: does tEIC have an effect on behavioral performance in the first place?

The results of Simulations A, B, and C (see Text S2 and Fig. S1 for Simulations B and C) suggested that the setting of the first tEIC experiment should be similar to that of Simulation A (Fig. 4(d)) for the following reasons. The tEIC intracellular effect was expected to be more pronounced in a setting like Simulation A in comparison with the other simulations having the same resistance intervals from the separator. If, on the other hand, the tEIC channels were conventional EEG channels (namely, as in Simulations B and C), the tEIC intercellular effect would vary from one EEG generator to the other.

**Figure 5:**
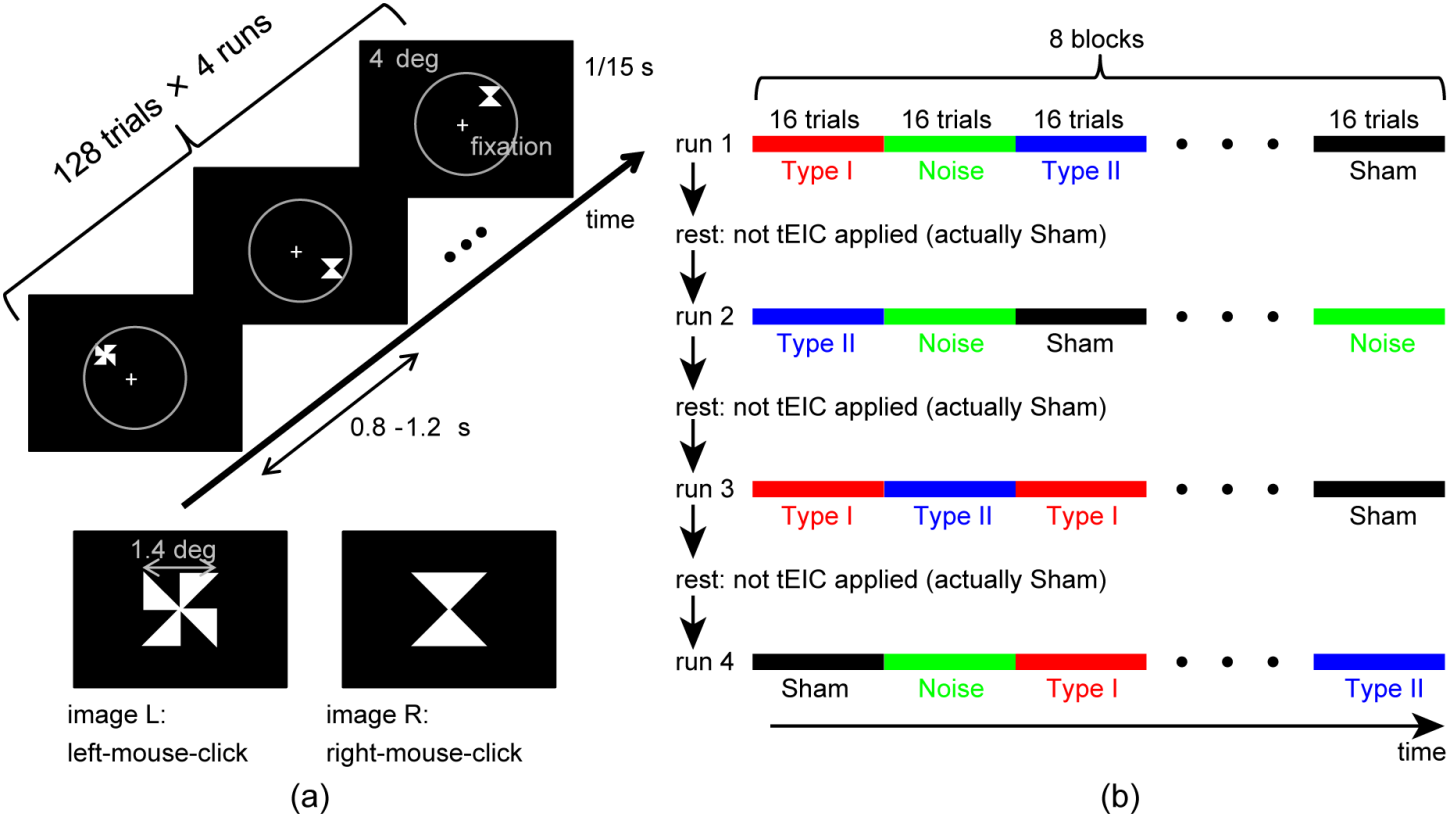
Behavioral experiment. A selective response (mouse-click) task for visually presented stimuli was performed. (a) Top: Visual stimulus sequence. Each stimulus is presented for a duration of 1/15 s with a stimulus onset asynchrony (SOA) of 0.8–1.2 s at a random location along the four-degree visual angle circle (the circle was invisible). Bottom: Images L and R for the selective mouse-click. (b) A total of 512 trials (128 trials×4 runs) were divided into 32 blocks (16 trials for each block). The tEIC conditions, Sham, Type I, Type II, and Noise, were randomly switched every block (the Noise condition was excluded from further analyses due to electronics problems). Note that tEIC was simultaneously (not in a before-and-after manner) applied to the behavioral experiment.

A selective response task for visually presented targets (top panel in Fig. 5(a)) was conducted. This task consisted of 128 trials for each of four runs (Fig. 5(b)). In each trial, one of two images, L and R (bottom panel in Fig. 5(a)), was randomly chosen and presented at a random location that was in internal contact with a circle (the circle was invisible) having the four-degree visual angle around the fixation cross for 1/15 s (four frames at a frame rate of 60 Hz). The L and R images were each constructed from four isosceles right triangles with a very low intensity of 420 cd/m^2^. The stimulus onset asynchrony (SOA) was randomly set to a very short duration of 0.8–1.2 s. The stimuli were presented with a visual stimulator (ViSaGe, Cambridge Research Systems) and an LCD projector (X700, Canon). The subjects were instructed prior to the experiment to left-mouse-click with their right index finger (right-mouse-click with their right middle finger) as soon as they saw image L (R). The very dark stimuli and very fast SOA meant that the subjects had to perform a somewhat challenging response task; we wondered if the reaction times would be modulated with tEIC.

During the behavioral experiment, four different conditions, namely Sham, Type I: separator +2.9 (mean) ±1.1 (standard deviation) kΩ, and Type II: separator –3.9±1.3 kΩ, and Noise, were applied between EEG electrode F3 (near the prefrontal cortex) and the right earlobe (an indifferent reference). We chose F3 only because this selective response task was a motor learning task and required right finger clicks. This setup was similar to that of Simulation A in that one of the tEIC channels was an indifferent reference. The experiment was divided into four runs separated by rest periods (Fig. 5(b)). Each run consisted of eight blocks, and each block consisted of 16 trials for a tEIC condition. tEIC was thus block-wise randomly applied in a simultaneous manner rather than a before-and-after manner.

The circuit in Fig. 3(b) or 3(d) was used for Type I, whereas the circuit in Fig. 3(a) or 3(c) was used for Type II; which one was used for each condition depended on development of electronics. We set the Type II resistances farther from the separators than the Type I resistances (a 1 kΩ additional margin) for the following reason. We were concerned about the increase in the impedance between the EEG electrodes with time. This directly indicated that the separator got closer to the Type II resistor, and thereby, the current to the brain hyperbolically increased (to the point of saturating at ∼1 mA). Meanwhile, we actually applied the Noise condition, which was just like a very weak current tRNS comparable to tEIC currents (6.3 nA rms), as a control experiment. However, we realized that we had not set the Noise condition equally for all the subjects due to problems regarding the electronics for the condition (only 0.63 nA rms was applied to two subjects). Therefore, we excluded the Noise condition from further analyses.

A monopolar 31-channel EEG (extended international 10–20 system) was simultaneously recorded during the behavioral experiment with the conventional amplifier settings (reference: right earlobe (a tEIC channel), ground: nose, passband: 0.5–100 Hz, gain: 1000 for Fp1 and Fp2, 2500: the other channels); i.e., the Type I (Type II) circuit was simply attached to F3 and the reference in parallel for the Type I (Type II) condition.

### Participants

Sixteen healthy subjects participated in the experiment (age: 25.1 (mean) ±6.9 (standard deviation); female: 1; left-handedness: 1; education: 8 (undergrad), 6 (grad), 2 (Ph. D)). The subjects gave written informed consent after they were given a detailed explanation of this study. The study received approval from the Ethics Committee for Human and Animal Research of the University of Tokyo.

### Results: EEG Waveforms

We excluded two out of 16 subjects from further analyses since they exhibited the Type I polarity inversion in other channels besides F3, so we decided that tEIC did not work properly in their cases (Fig. S2). Trials corresponding to missed, too early, and too late button pressings were eliminated from the analysis. Trials showing EEG blinking artifacts and electrical noise from switching transients were also excluded. If a researcher looks for EEG blinking artifacts in an EEG at Fp1 or Fp2 (near the eyeballs), s/he may have to set a threshold value for each tEIC condition. The EEG magnitude of Type I (Type II) for any channel was almost always smaller (larger) than that of the Sham condition, because the Type I (Type II) resistor attached to an EEG channel and the reference (like the settings of Simulation A) decreased (increased) the impedance between any EEG channel and the reference. Therefore, tEIC resistors worked as an additional EEG amplifier gain, and Type I (Type II) depressed (enhanced) EEG blinking artifacts. Consequently, only valid EEG trials (trigger: stimulus onset, pre-trigger duration: 0.1 s, and post-trigger duration: 0.5 s) were extracted.

**Figure 6:**
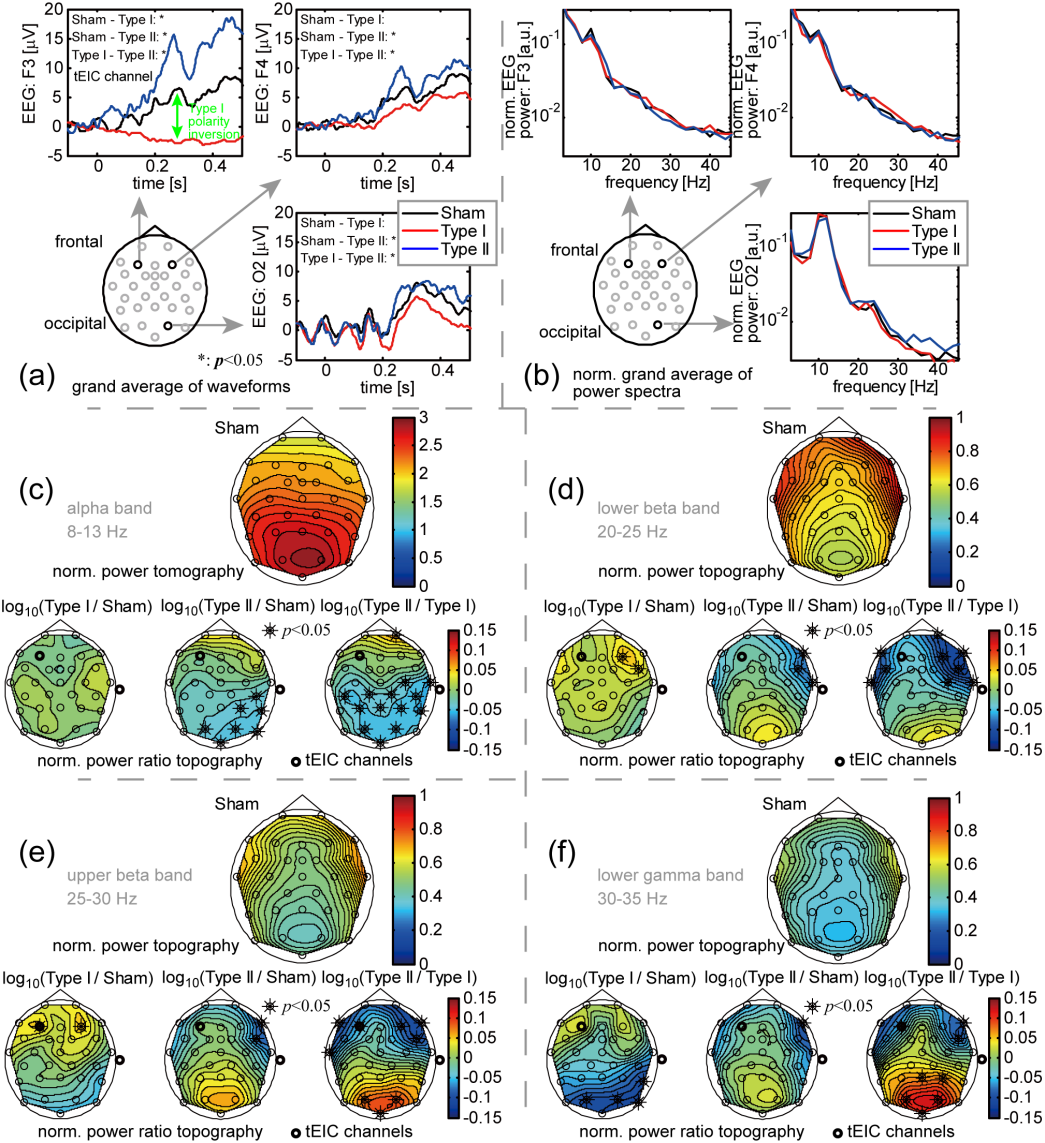
EEG results of the tEIC-applied behavioral experiment. (a) tEIC-conditional EEG grand averages at EEG channels F3 (tEIC channel), F4, and O2 for 14 validated subjects (duration: from −0.1 to 0.5 s, stimulus onset: 0 s). The averaged magnitudes for the post-trigger duration (from 0 to 0.5 s) were statistically tested (*: significant difference among the averaged magnitudes, *p<*0.05 with Bonferroni correction; see Fig. S3 for all the channels). These grand averages were consistent with the expectations with reference to Ho-Thevenin’s theorem. (b) Normalized EEG grand power spectrum averages for the post-trigger duration (not the power spectra of the EEG grand averages). Type I at F3 and F4 and Type II at O2 showed temporal-high-pass-filter-like characteristics. Since tEIC was designed not to have any frequency response above 4 Hz, this would be caused by spatial frequency responses. (c) Alpha band (8–13 Hz) results. Top: Topography of the normalized EEG grand power averages for the post-trigger duration for Sham; Bottom: Ratios of the normalized grand power averages for the post-trigger duration on a logarithmic scale: log_10_(Type I/Sham), log_10_(Type II/Sham), and log_10_(Type II/Type I) (*: significant difference among the normalized power averages, *p<*0.05 with Bonferroni correction). (d) Lower beta band (20–25 Hz) results. (e) Upper beta band (25–30 Hz) results. (f) Lower gamma band (30–35 Hz) results. The presentation styles of (d)–(f) are the same as in (c). The EEG generators of the alpha band seemed to be densely distributed in the occipital area, whereas those of the other bands seemed to be densely distributed in the frontal area (top panels in (c)–(f)). Type I enhanced (depressed) the power of the area in which the EEG generators were densely (sparsely) distributed, whereas Type II enhanced (depressed) for the area in which the EEG generators were sparsely (densely) distributed (bottom panels in (c)–(f)). These results imply that tEIC works as a spatial filter (Type I: spatial high-pass filter, Type II: spatial low-pass filter) due to the synergetic tEIC effect; i.e., Type I differentiates and Type II merges EEG generator activities.

Figure 6(a) shows the EEG averages at F3 (tEIC channel), F4, and O2 over the validated trials and subjects for Sham, Type I, and Type II (the so-called grand average). Baseline zeroing was done for the pre-trigger duration. The significance of the averaged magnitude over the post-trigger duration (from 0 to 0.5 s in latency, the EEGs of other conditions were contaminated in the pre-trigger duration) among Sham, Type I, and Type II conditions was assessed by a one-way repeated measures of ANOVA for each channel (*p*<0.05). The results of a post hoc multiple comparison test with the Bonferroni correction (*p*<0.05) are displayed in each panel (see Fig. S3 for all the channels). Note that the signed (not the absolute value of) EEG averages were statistically tested since all the channels except Type I at F3 showed almost completely positive-definite waveforms and this exception was useful for checking the Type I polarity inversion. The Type I polarity inversion was only observed at F3, and moreover, the magnitude order, namely Type I ≤ Sham ≤ Type II, was significant at F3. The same magnitude order was observed in the other channels (Type I ≤ Sham: tendency, Type I ≤ Type II and Sham ≤ Type II: significant). Only Type II showed significance because it probably worked as an additional EEG amplifier gain for not only the tEIC channel but also the other channels. This was the same phenomenon as we described for thresholding of EEG blinking artifacts. Thus, tEIC seemed to work properly at a level at which Ho-Thevenin's theorem held. Note that the P100 visual response was invisible probably because very dark stimuli were presented in randomly selected areas of the visual field and the alpha-band oscillation at O2 for a duration from –0.1 to 0.1 s, which was also observed in other occipital channels (Fig. S3), would be residual alpha-ringing [46] after averaging.

Figure 6(b) shows the normalized EEG grand power spectrum averages (not the power spectra of the EEG grand averages) of these three channels over the post-trigger duration under the tEIC conditions. The normalization was done by division of the sum of powers from 4 Hz (to avoid the influence of low-frequency fluctuations) to 45 Hz (to avoid the influence of power-line noise at 50 Hz) for each channel and each condition. The normalization is needed in order to make conditional comparisons to avoid the above-mentioned additional EEG amplifier gain. Now, Type I exhibited power higher than or equal to Sham did for the frequency band above 20 Hz at F3 (a frontal channel). Type I acted just like a temporal high-pass filter. A similar characteristic was observed at F4 (another frontal channel). On the other hand, Type II acted like a temporal high-pass filter at O2 (an occipital channel). Here, we must stress that the tEIC circuits were designed not to have any temporal frequency response above 4 Hz. Therefore, these temporal-filter-like characteristics were probably inherited from the spatial-filter characteristics of tEIC. Indeed, it is widely known that a spatial filter occasionally plays a temporal filter role in EEG analyses because the spatial distributions of EEG generators have a temporal frequency dependence [4].

We investigated the spatiotemporal characteristics of all of the channels. The top row of Fig. 6(c) shows a topological map of the normalized EEG grand power average of the 8–13 Hz (alpha) band for Sham, which should be treated as raw, or not spatially filtered, data. The spatial power peak of this band was located in the occipital area, and hence, the EEG generators of this frequency band seemed to be densely distributed in that area. The top rows of Figs. 6(d)–6(f) show topological maps of 20–25 (lower beta), 25–30 (upper beta), and 30–35 Hz (lower gamma) for Sham, which should also be treated as raw data. These maps indicate that the EEG generators in these bands were densely distributed in the frontal area.

The bottom rows of Fig. 6(c) show topological maps of the ratios of the normalized EEG grand power averages on a logarithmic scale: namely, log_10_(Type I/Sham), log_10_(Type II/Sham), and log_10_(Type II/Type I) for the alpha band (positive: red, negative: blue). The significance of the grand power averages (not on a logarithmic scale) among the Sham, Type I, and Type II conditions was assessed by a one-way repeated measures of ANOVA for each channel (*p*<0.05). The asterisks indicate the results of a post hoc multiple comparison test with the Bonferroni correction (*p*<0.05). Type I depressed the power of this frequency band for the frontal area and enhanced it for the occipital area. This was just a tendency and was not statistically significant. On the other hand, Type II exhibited the opposite characteristics for this frequency band (7 out of 31 channels showed significance). The opposing characteristics of Type I and Type II were also evident in the maps of log_10_(Type II/Type I) (14 out of 31 channels showed significance). The bottom rows of Figs. 6(d)–6(f) show the same topological maps as those of Fig. 6(c) but for the lower beta, upper beta, and lower gamma bands. The topological maps of these three frequency bands present the opposite spatial enhancement and depression characteristics compared with those of the alpha band, i.e., for log_10_(Type I/Sham): frontal enhancement and occipital depression; and for log_10_(Type II/Sham): frontal depression and occipital enhancement. Some channels showed significance. Thus, the two-factor ((Type I, Type II) × (the alpha band, the other bands)) interaction effects (enhancement, depression) on the normalized EEG grand power averages were very contrasting.

### Discussion: EEG Waveforms

The two-factor interaction effects seem to be reasonable considering the spatial-filter-like characteristics of tEIC. Let us recall the expected EEG observations of the synergetic tEIC effect pointed out in Simulation A; i.e., Type I works as a spatial high-pass filter, and Type II works as a spatial low-pass filter. If we assume that, in the settings of Simulation A (Figs. 4(d) and 4(e)), *E*_1_ is the lower beta, upper beta, or lower gamma EEG generator, *E*_2_ is the alpha band one, channel α is F3, and channel β is another channel, then the analyses based on the normalized power of the EEG data (Figs. 6(c)–6(f)) and those of the Simulation A by using *p*_1_/*q*_1_ and *p*_2_/*q*_2_ would bear a strong resemblance to each other. If, in fact, we subtract log_10_(Type I/Sham) for the lower beta, upper beta, or lower gamma band from log_10_(Type I/Sham) for the alpha band and do so as well for log_10_(Type II/Sham), the calculations will be essentially the same as those of *p*_1_/*q*_1_ and *p*_2_/*q*_2_ in Simulation A (division is turned into subtraction by taking the log) and the actual calculations will obviously result in an enhancement of the red and blue contrast in the topological maps of the ratios. This implies that the synergetic tEIC effect actually occurred in the experiment.

Let us evaluate the spatial influence of tEIC. This actually depends on how application of tEIC changes the resistance seen from an electric source in the brain. If it is big, the source is well modulated. Now, the resistance is roughly estimated as follows. In the case that a current dipole ***j***, or an EEG generator, is located at ***r***_0_ in the brain, we observe a voltage potential *V* at ***r*** approximately as *V* ∝ ***j***⋅(***r***–***r***_0_)/|***r***–***r***_0_|^3^ where “⋅” denotes the scalar product. Comparing this and the scalar case of Ohm’s law *V* = *jR*, the resistance *R* ( =  ***j*** **⋅**(***r***–***r***_0_)/|***r***–***r***_0_|^3^/|***j***|) depends on not only distance ***r***–***r***_0_ but also the angle between ***j*** and ***r***–***r***_0_. We can only say that the spatial influence of tEIC depends on sensitivity, or 1/*R*. A similar discussion applied to the non-tEIC channel in the simulation. That is, tEIC has a significant influence on distant electric sources in some situations. Thus, it would not be strange that tEIC applied to a frontal area modulated an occipital area, as shown in Fig. 6.

We would like to stress that the spatial-filter-like tEIC effects are definitely different from the spatial filters in the signal processing sense (e.g., a Laplacian filter). tEIC actually modulated EEG generators such that the EEG observations changed as if they were spatially filtered. The spatial-filter-like tEIC effects are somewhat similar to the high-definition tDCS [21]–[24] as far as their having actual spatially dependent effects on EEG generators, but the tEIC has not only spatially dependent but also temporally dependent effects because it has in principle no intracellular effect on the EEG-silent EEG generators.

We have evaluated electrophysiological effects of tEIC by examining EEGs. Here, one may think that MEP would be more suitable for such an evaluation. However, the effects of tEIC cannot be evaluated with MEP. The reason is that if an MEP measurement is conducted simultaneously with tEIC, tEIC enhances and depresses the MEP-induced current in the MEP measurement, which is much larger than the extracellular EEG currents. Therefore, MEP never reflects the true tEIC effects. Once again, we should stress that tEIC is not a constant current source, but a (negative) resistor.

### Results: Behavioral Performances

**Figure 7:**
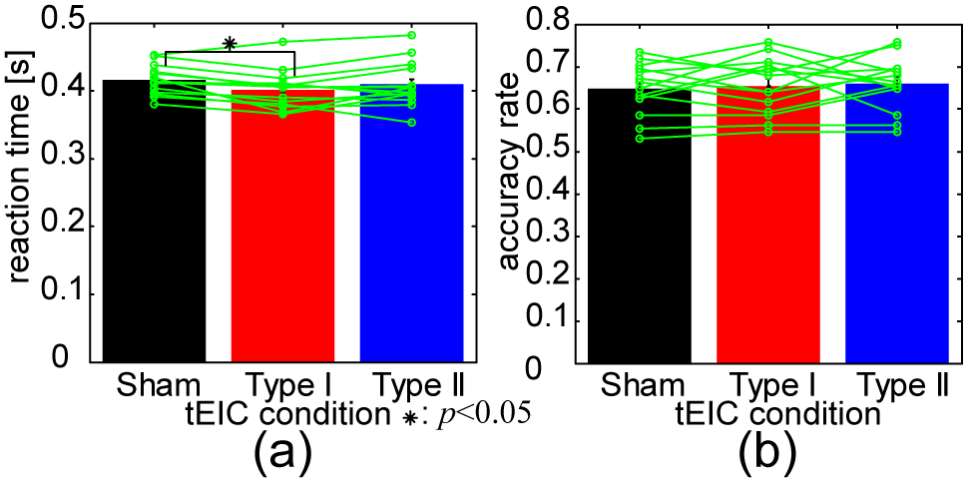
Behavioral results of the tEIC-applied behavioral experiment. (a) tEIC-conditional means of reaction times for 14 validated subjects. Individual reaction times are superimposed on these results. Type I showed a significant improvement effect in reaction time compared with Sham (*p<*0.05 with Bonferroni correction). (b) tEIC-conditional means of accuracy rates over the subjects. The presentation style is the same as in (a). No significant differences were observed.

The significance of the reaction time differences among the Sham, Type I, and Type II conditions was assessed by a one-way repeated measures of ANOVA for the validated trials and subjects (*F*(2,26) = 4.68, *p*<0.05). Figure 7(a) shows the mean reaction times of the subjects as a bar graph, the individual reaction times (green plots), and the results of a post hoc multiple comparison test with the Bonferroni correction (black line with asterisk). Only Type I significantly improved reaction times from Sham (*p*<0.05). The accuracy rates are displayed in the same style in Fig. 7(b), but there were no significant accuracy rate differences among these conditions (*F*(2,26) = 0.273, *p*>0.05).

### Discussion: Behavioral Performances

Since we asked the subjects to push a button as soon as possible, the significance and non-significance of these results would be merely caused by the tradeoff between speed and accuracy. If tEIC were applied to a different behavioral experiment focused on accuracy, it is quite possible that tEIC would be able to modulate the accuracy rate. It would depend on the experimental design whether an electric brain modulation technique such as tDCS, tRNS, tACS, and tEIC affects the reaction time or other performance aspects such as the accuracy rate, success rate, or perception. Indeed, limited to simultaneous applications of tDCS and tACS to behavioral experiments (because tEIC is simultaneous application), some applications affected reaction time [14], [34], while others affected other performance aspects [15], [18], [28], [30]. Thus, they were exclusive. We would like to investigate how tEIC affects the accuracy rate in the near future.

When the excluded Noise condition was included, the ANOVA for the reaction times also exhibited significance (*F*(3,39) = 3.10, *p*<0.05) and the ANOVA for the accuracy rate did not exhibit significance (*F*(3,39) = 0.150, *p*>0.05) (Fig. S4). A post hoc multiple comparison test of the reaction times with the Bonferroni correction showed significant improvements for Type I compared with Sham and Noise (*p*<0.05). Since the Noise condition was not set equally for the subjects, we cannot claim anything about the reaction time improvement between Type I and Noise. However, the exclusion of the Noise condition seemed to have no influence on the results with the Noise condition excluded.

Let us discuss the significant facilitation effect on the reaction times for Type I. In principle, Type I enhances not only EPSPs but also IPSPs. However, since the equilibrium potential of EPSPs is farther from the membrane resting potential than that of IPSPs [47], the EEG generators seem to be mainly EPSPs. Therefore, the tEIC circuit, which works with reference to the real-time EEG, probably affects the EPSPs accordingly. Although the relationship between the tEIC intercellular effect of Type I and behavioral performances is unclear, the behavioral modulation that occurs through the tEIC intercellular effect may be smaller than through the tEIC intracellular effect because of the opposite effects of the inward and outward currents on the membrane potentials (Fig. 1(b)). The tEIC linear effects may in turn cause nonlinear effects, but do the nonlinear ones systematically cancel the linear ones? If they did, the EEG results would not be consistent with a simulation based on linearity. Thus, we speculate that the real-time enhancement of EPSPs with Type I simply improved reaction times in this behavioral experiment.

As the discussion of the EEG waveform indicates, Type I and Type II had opposite tEIC intra- and inter-cellular effects (Fig. 6). However, the tEIC behavioral effects were not so; i.e., Type I significantly facilitated the behavioral performance in this experiment but Type II did not significantly inhibit it. This might not be so unnatural because the cathodal tDCS, which is electrically opposite to the anodal tDCS, often does not exhibit behavioral significance compared with the sham condition, while the anodal tDCS does so compared with the sham condition [6], [8], [11], [15]. A speculated reason is that the resistor interval from the separator in Type II was not symmetrical to that in Type I due to the additional 1 kΩ margin from the separator. This implies that Type II relatively had less influence on the behavioral performance than Type I had. Indeed, regarding the influence on EEG, each normalized power ratio topography of log_10_(Type II/Sham) for the lower beta, upper beta, and lower gamma bands was somewhat more similar to each corresponding normalized power topography of Sham than each corresponding normalized power ratio topography of log_10_(Type I/Sham) (Figs. 6(d)–6(f)), implying that Type II did not so much change the shape of the topographies of Sham compared with Type I. Meanwhile, the direct, or even statistical, reason for the lack of significance of Type II must be that the individual mean reaction times of Type II seemed to be divided into facilitation and inhibition groups compared with those of the Sham condition (Fig. 7(a)). Thus, Type II might have complicated effects like those of tACS [37]. In any case, further experimental investigations are needed.

We applied tEIC while the participants were performing a behavioral task; i.e., the usage was in not a before-and-after manner, but a real-time one. Let us discuss the prolonged effects of tEIC. The tEIC conditions were block-wise changed, and each block consisted of 16 trials (an average of 16 s, see Fig. 5(b)). The behavioral significance changed every 16 s of sampling. Each run consisted of 8 blocks (average of 128 s), and the mean rest time between the runs was 350 s. The prolonged effects in this experiment would be very short compared with the block duration, and so the rest time was long enough to avoid any prolonged effects from the previous run. However, because of the limited size of this study, further investigations into the possible prolonged effects of tEIC are necessary.

Finally, let us briefly compare tEIC with other forms of current stimulation. tDCS and tACS with a sub-mA-order current had almost no effect [32], [48], [49], except in a recent study [18]. Since the EEGs with Type I were at most 10 µV (Fig. 6(a)) and the resistors of Type I were at least 1 kΩ (actually –1 kΩ), the resulting current flowing through the resistor was estimated to be at most 10 nA. Despite it having such a very tiny current, Type I significantly improved reaction times. The current of tEIC is the result of changes in the voltage of the EEG generators, whereas the current of the various current stimulations changes the voltages of the EEG generators (actually, it would affect synaptic plasticity). tEIC is by no means a current stimulation and employs a negative resistor with an absolute value of a few kΩ, which is much smaller than the typical input impedance of EEG recordings and moreover goes into the negative resistance domain. Thus, tEIC and the current stimulations should not be compared in terms of their current, just as they cannot be compared in terms of their resistance.

In summary, we proposed tEIC and showed how it could be made to affect real-time EEG observations and behavioral performance. We conclude that tEIC, at least Type I, can modulate behavioral performance like tDCS, tRNS, and tACS can. The doorway to tEIC research has been opened, and we hope that tEIC will be used as another technique to modulate behavioral performance.

## Supporting Information

Figure S1 **Simulations of tEIC.**
(PDF)

Figure S2 **EEG averages of two invalid subjects.**
(PDF)

Figure S3 **Grand average of waveforms for all the channels.**
(PDF)

Figure S4 **Behavioral results of the tEIC-applied behavioral experiment with the excluded Noise** **condition included.**
(PDF)

Text S1 **Workarounds to ensure stable operation of the electronics in** **Fig. 3** **.**
(PDF)

Text S2 **Results and discussion for Simulations B and C.**
(PDF)
