# Supplementary material for: Transcranial Extracellular Impedance Control (tEIC) Modulates Behavioral Performances
Source: PLoS One. 2014 Jul 21;9(7):e102834. doi: 10.1371/journal.pone.0102834 (PMC4105436; doi:10.1371/journal.pone.0102834)
Supplement: Figure S1 — Simulations of tEIC. (PDF) [file pone.0102834.s001.pdf]

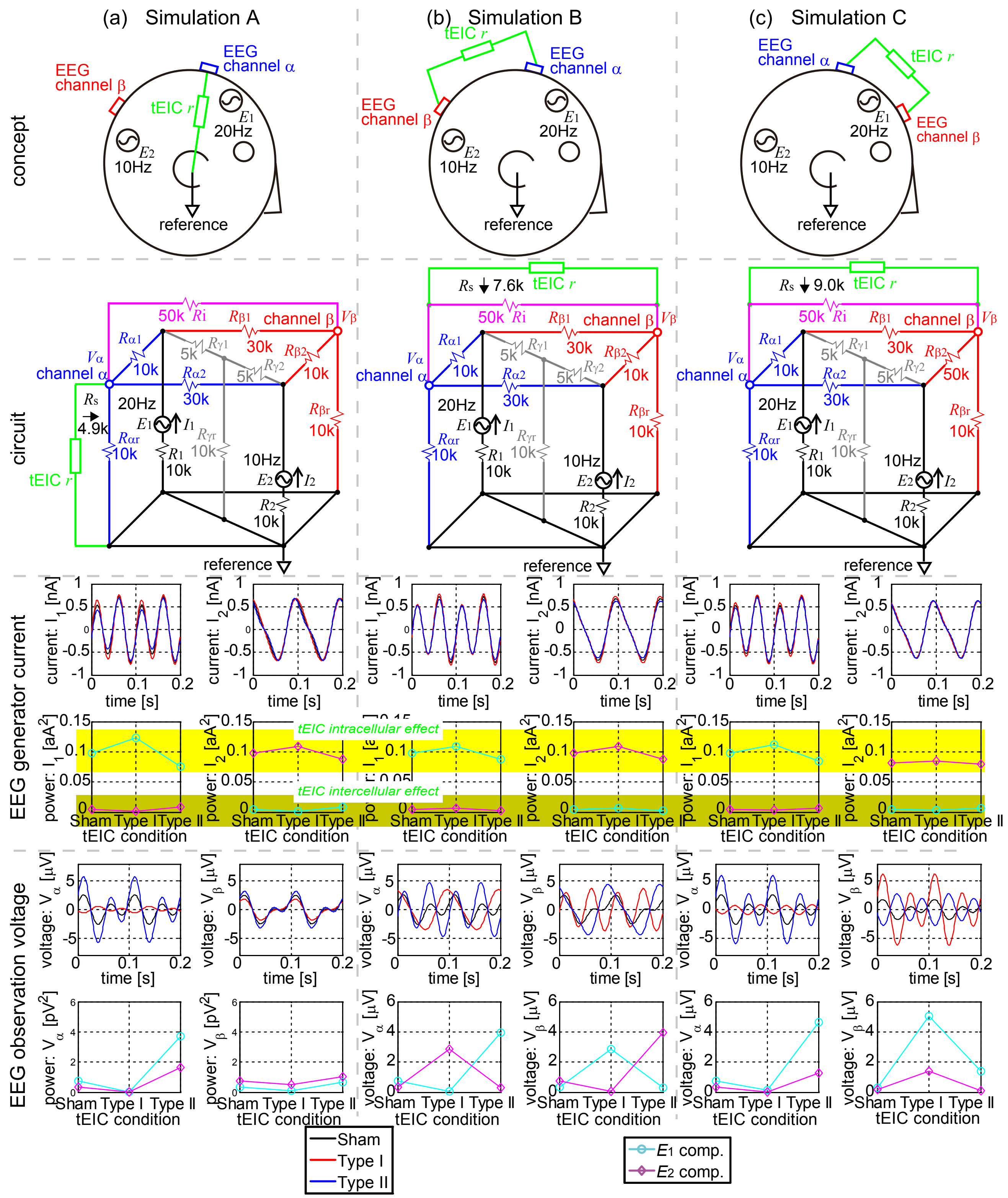

(a) Simulation A
(b) Simulation B
(c) Simulation C
concept
circuit
EEG generator current
EEG observation voltage
EEG channel α
EEG channel β
tEIC r
E1 20Hz
E2 10Hz
reference
channel α
channel β
Rs 4.9k
Rs 7.6k
Rs 9.0k
50k Ri
tEIC intracellular effect
tEIC intercellular effect
time [s]
tEIC condition
Sham Type I Type II
Sham
Type I
Type II
E1 comp.
E2 comp.

Figure S1. Simulations of tEIC. The simulation conditions are column-wise the same. (a) Simulation A. Top Row: A tEIC resistor was connected between an EEG channel and the indifferent reference. Second Row: Simulation circuit. gray T-circuit: inter connection of two EEG generators E_1 (20 Hz sine) and E_2 (10 Hz cosine), blue T-circuit: EEG observation on EEG channel α, red T-circuit: EEG observation on EEG channel β, and magenta connection: direct path between EEG channels α and β. EEG channel α was set to be more sensitive to E_1 while EEG channel β was set to be more sensitive to E_2. Third Row: tEIC-conditional current waveforms of the EEG generators (top row) and their powers resolved into E_1- and E_2-originated currents (bottom row). Type I enhanced and Type II depressed the intrinsic currents (E_1-originated current in I_1 and E_2-originated current in I_2) compared with the Sham condition. This is the tEIC intracellular effect. Type I depressed and Type II enhanced the interference currents (E_2-originated current in I_1 and E_1-originated current in I_2) compared with the Sham condition. This is the tEIC intercellular effect. Thus, the synergetic tEIC effect clearly occurred; i.e., Type I differentiated and Type II merged the EEG generator activities. Bottom Row: tEIC-conditional voltage waveforms of the EEG observations (top row) and their powers resolved into E_1- and E_2-originated voltages (bottom row). The results in the top left panel showed that the tEIC-conditional waveform changed on the basis of Ho-Thevenin’ s theorem. (b) Simulation B. The same simulation as (a) except that the tEIC channels were an E_1-sensitive channel (EEG channel α) and an E_2-sensitive channel (EEG channel β). The row-wise presentation styles are the same as in (a). Although the tEIC intracellular effect has almost the same characteristics as in (a), the tEIC intercellular effect was inverted between Type I and Type II compared with (a). (c) Simulation C. The same simulation as shown in (b) except that the tEIC channels were two E_1-sensitive EEG channels. The tEIC intra- and inter-cellular effects have almost the same characteristics as in (a). The tEIC resistor was set + 4 kΩ from the separator for Type I and – 4 kΩ from the separator for Type II for all the simulations.
