# Supplementary material for: Transcranial Extracellular Impedance Control (tEIC) Modulates Behavioral Performances
Source: PLoS One. 2014 Jul 21;9(7):e102834. doi: 10.1371/journal.pone.0102834 (PMC4105436; doi:10.1371/journal.pone.0102834)
Supplement: Figure S2 — EEG averages of two invalid subjects. (PDF) [file pone.0102834.s002.pdf]

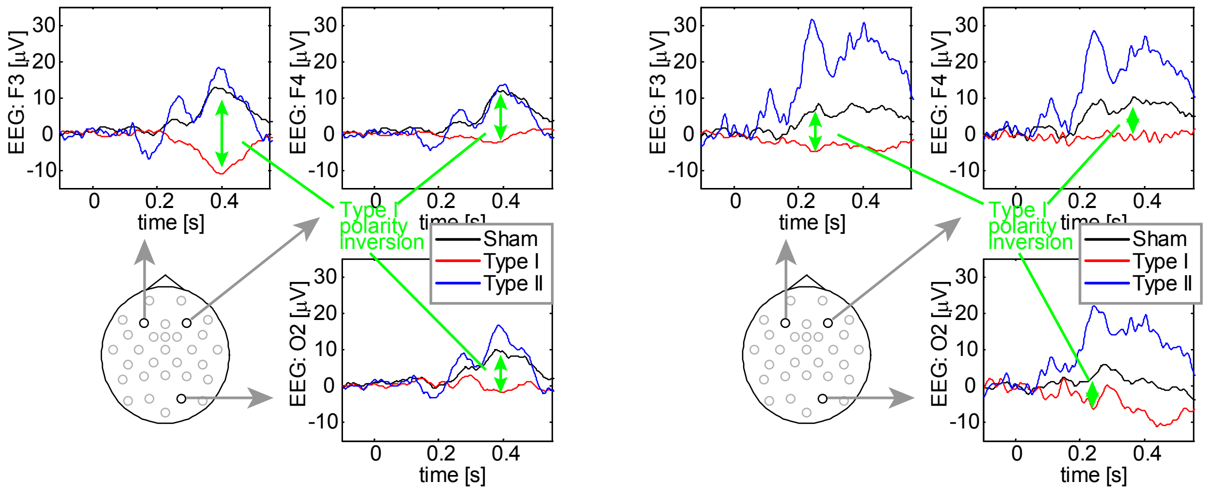

Figure S2. EEG averages of two invalid subjects. The Type I polarity inversion was observed on other channels in addition to F3 (tEIC-channel), and it is doubtful that tEIC worked as a current stimulation. Thus, these two subjects were excluded from further analysis.
