# Supplementary material for: Transcranial Extracellular Impedance Control (tEIC) Modulates Behavioral Performances
Source: PLoS One. 2014 Jul 21;9(7):e102834. doi: 10.1371/journal.pone.0102834 (PMC4105436; doi:10.1371/journal.pone.0102834)
Supplement: Figure S3 — Grand average of waveforms for all the channels. (PDF) [file pone.0102834.s003.pdf]

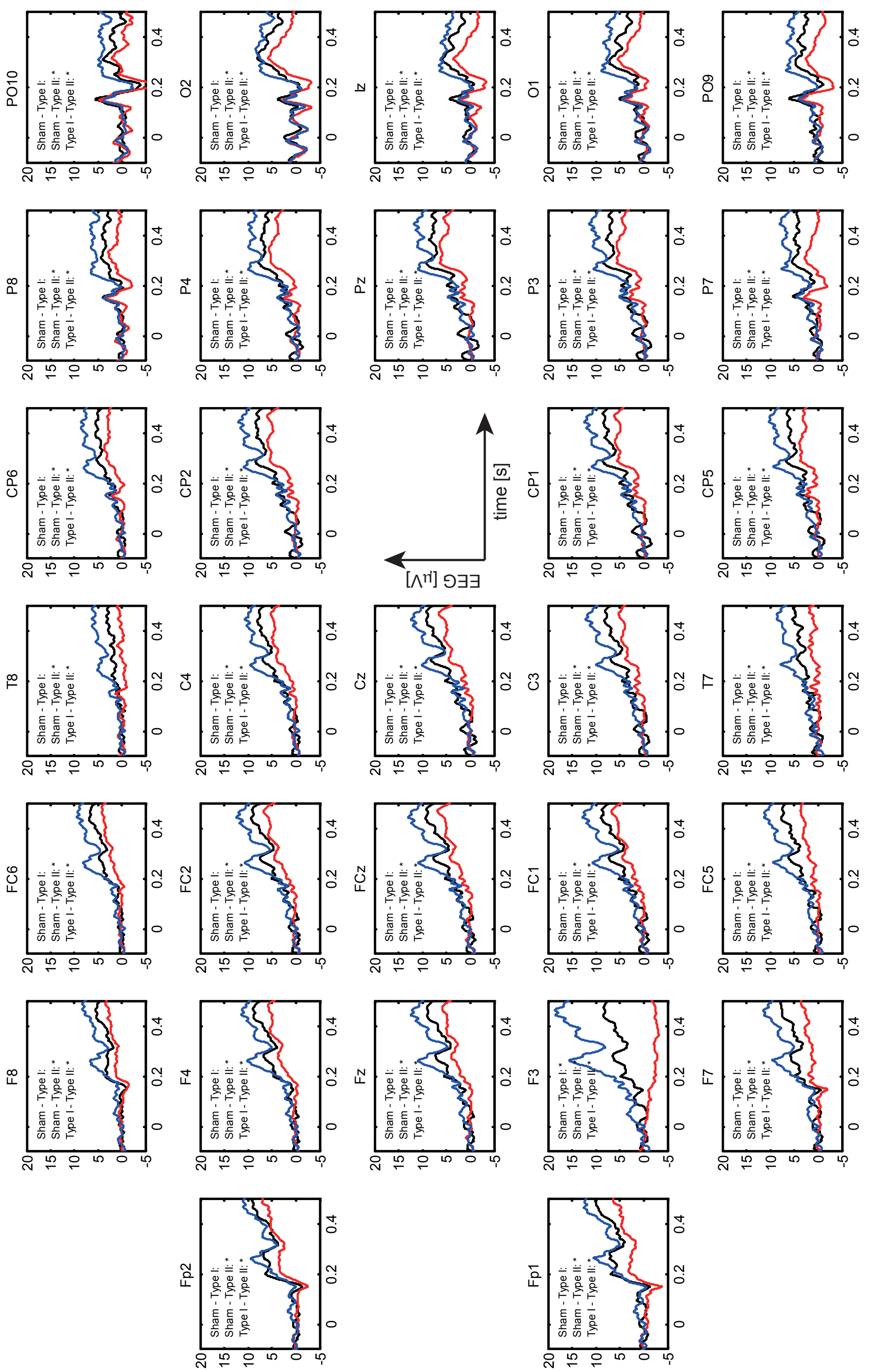

Figure S3. Grand average of waveforms for all the channels.

Black: Sham, red: Type I, and blue: Type II. tEIC channel: F3.

*: significant difference among the averaged magnitudes, $p < 0.05$ with Bonferroni correction
