# Supplementary material for: Transcranial Extracellular Impedance Control (tEIC) Modulates Behavioral Performances
Source: PLoS One. 2014 Jul 21;9(7):e102834. doi: 10.1371/journal.pone.0102834 (PMC4105436; doi:10.1371/journal.pone.0102834)
Supplement: Figure S4 — Behavioral results of the tEIC-applied behavioral experiment with the excluded Noise condition included. (PDF) [file pone.0102834.s004.pdf]

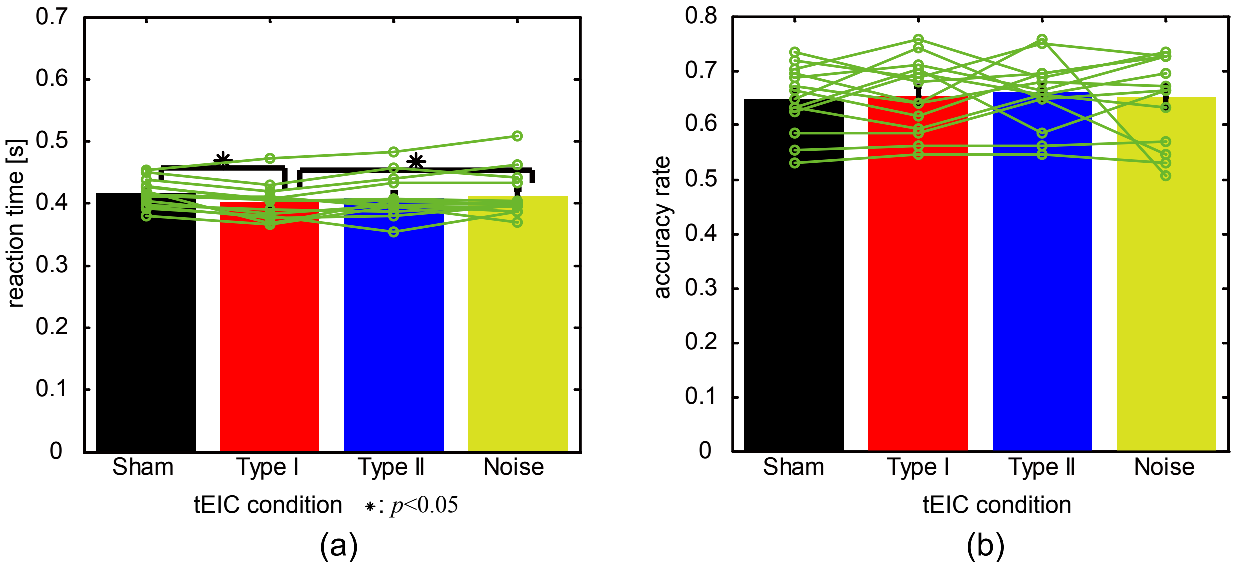

Figure S4. Behavioral results of the tEIC-applied behavioral experiment with the excluded Noise condition included. (a) Reaction time result. (b) Accuracy rate result. The significant improvement of the reaction times of Type I compared with Noise was meaningless because the Noise condition was not equally set due to an electronics problem. If this effect is neglected, the exclusion of the Noise condition seemed to have no influence on the results without the Noise condition. We originally intended to experimentally show that the Noise condition, or very low current tRNS (6.3 nA rms), does not have any effect on behavioral performance. The fact would have been evidence that tEIC, or negative resistor attached to the scalp, has a different mechanism from that of current stimulation.
