# Supplementary material for: Transcranial Extracellular Impedance Control (tEIC) Modulates Behavioral Performances
Source: PLoS One. 2014 Jul 21;9(7):e102834. doi: 10.1371/journal.pone.0102834 (PMC4105436; doi:10.1371/journal.pone.0102834)
Supplement: Text S1 — Workarounds to ensure stable operation of the electronics in Fig. 3 . (PDF) [file pone.0102834.s005.pdf]

Supporting Text 1

Each following workaround is only necessary in the case that a problem with the electronics occurs. A small capacitor (say, 220 pF) should be connected in parallel with every Rc that is between the output and inverted input terminals of OP1-OP4 in order to prevent higher frequency oscillations. A small laminated ceramic capacitor (say, 0.1 μF) should be connected in parallel with every electrolytic capacitor Cc to reduce switching transients. A make-before-break (MBB) switch, which connects both terminals at the moment of switching, should be used for all the switches except SW4, whereas a break-before-make (BBM) switch, which is a normal switch and disconnects both terminals at the moment of switching, should be used for SW4. A semiconductor switch is better choice (no acoustical switching noise) for any of the switches but it must be carefully selected from among the various complementary metal-oxide semiconductor (CMOS) switches which have a very low leakage current (say, 10 pA). Note that we treat nA-order currents. Unfortunately, such an MBB switch may not be available (due to end of life), and hence a mechanical MBB switch should be used instead. In this case, one must take care to ensure that a subject does not hear switching sounds.
