# Supplementary material for: Transcranial Extracellular Impedance Control (tEIC) Modulates Behavioral Performances
Source: PLoS One. 2014 Jul 21;9(7):e102834. doi: 10.1371/journal.pone.0102834 (PMC4105436; doi:10.1371/journal.pone.0102834)
Supplement: Text S2 — Results and discussion for Simulations B and C. (PDF) [file pone.0102834.s006.pdf]

Supporting Text 2

Simulation A

Figure S1(a), which is the settings and results of Simulation A, contains the same information as in Fig. 4. This would be useful to compare results among the simulations.

Simulation B

The settings of Simulation B (top and second rows of Fig. S1(b)) were the same as in Simulation A except that the tEIC channels were channels α and β (Type I: –3.6 kΩ and Type II: –11.6 kΩ for R_s = 7.6 kΩ). The presentation in Fig. S1(b) corresponds to the one used in Fig. S1(a). While the tEIC intracellular effect in this simulation was smaller than the one in Simulation A for the same resistance interval from the separator (separator ± 4 kΩ), the same tEIC intracellular effect occurred (third row of Fig. S1(b)). However, the tEIC intercellular effect was inverted between Type I and Type II compared with Simulation A. This inversion is intuitively obvious because the tEIC worked as if two EEG generators had been connected to a Type I circuit (top row of Fig. S1(b)). Thus, Type I enhanced and Type II depressed the mutual current interferences. The EEG observations in this simulation (bottom row of Fig. S1(b)) are also intuitively obvious. That is, Type I resulted in merged waveforms and Type II differentiated ones. Note that the resistance versus current characteristic (middle panel in Fig. 2(b)) is useful for the intuitive comprehension here. When the Type-I negative resistor is placed between the two sites where the tEIC is attached, more current flows between these sites than in the case that the sites are shunted with a short circuit. As a result, the sites appear to be forced together. In contrast, the Type-II resistor forces the sites away from each other by pushing the current away from the sites.

Simulation C

The settings of Simulation C (top and second rows of Fig. S1(c)) were the same as those of Simulation B except that channels α and β both had higher sensitivity to E_1 than to E_2 (by setting $R_{\beta 2}$ = 50 kΩ). The location of channel β equivalently moved toward E_1, as illustrated in the top panel of Fig. S1(c). The tEIC resistor settings were changed accordingly (Type I: –5.0 kΩ and Type II: –13.0 kΩ for R_s = 9.0 kΩ). While the tEIC intracellular effect in this simulation was smaller than that in Simulation A for the same resistance interval from the separator, the same tEIC intra- and inter-cellular effects occurred (third row of Fig. S1(c)), meaning that the tEIC intercellular effect was inverted between Type I and Type II compared with Simulation B. This is intuitively obvious because the Type I resistor here worked as if the E_1-originated current had been confined (top row in Fig. S1(c)). Thus, Type I depressed and Type II enhanced the current interference. The EEG observations also reveal a side effect of this simulation (bottom row of Fig. S1(c)). Since channel α was electrically near both EEG generators, the currents from the generators mainly flowed into the reference via channel α. When Type I was attached to the interconnection between channels α and β, the resistance of the interconnection decreased and thereby the current between the channels increased. Consequently, Type I decreased V_α including the E_1- and E_2-originated voltages and increased V_β including both these voltages. Type II had the opposite effect.
